# Relationship between EGFR expression and subcellular localization with cancer development and clinical outcome

**DOI:** 10.18632/oncotarget.26727

**Published:** 2019-03-08

**Authors:** Ge Yan, Mohamed E.M. Saeed, Sebastian Foersch, Jose Schneider, Wilfried Roth, Thomas Efferth

**Affiliations:** ^1^ Department of Pharmaceutical Biology, Institute of Pharmacy and Biochemistry, Johannes Gutenberg University, Mainz, Germany; ^2^ Institute of Pathology, University Medical Center, Mainz, Germany; ^3^ Universidad Rey Juan Carlos, Facultad de Ciencias de la Salud, Móstoles, Spain

**Keywords:** biomarker, oncogene, pathological parameters, prognosis, survival

## Abstract

Epidermal growth factor receptor (EGFR) as a prevalent oncogene regulates proliferation, apoptosis and differentiation and thereby contributes to carcinogenesis. Even though, the documentation on its clinical relevance is surprisingly heterogeneous in the scientific literature. Here, we systematically investigated the correlation of mRNA to survival time and pathological parameters by analyzing 30 datasets *in silico*. Furthermore, the prognostic value of membrane-bound, cytoplasmic (mcEGFR) and nuclear expression (nEGFR) of EGFR was experimentally analyzed by immunohistochemical staining of 502 biopsies from 27 tumor types. We found that protein expression of EGFR showed better prognostic efficiency compared to mRNA, and that mcEGFR expression was positively correlated with nEGFR expression (*p* < 0.001). Unexpectedly, both mcEGFR and nEGFR expression were associated with low T stage (*p* < 0.001 and *p* = 0.004; respectively). Moreover, positive mcEGFR was significantly related to high differentiation (*p* = 0.027). No significant correlation was found with any other pathological parameters. Collectively, our results imply that the oncogenic function of EGFR may be more related to nascent stages of carcinogenesis than to advanced and progressive tumors, which may as well explain at least partially the occurrence of secondary resistance against EGFR-directed therapy.

## INTRODUCTION

Epidermal growth factor receptor (EGFR), also known as ErbB1 or HER1, together with three homologues (HER2, HER3 and HER4) composes the ErbB family of tyrosine kinase receptors (TRKs). EGFR represents a transmembrane receptor with a molecular weight of 175 kDa. Upon binding to its ligands such as epidermal growth factor (EGF) or transforming growth factor-α (TGF-α), EGFR homo- or hetero-dimerizes with its counterparts [1]. Such dimerization stimulates auto-phosphorylation of several tyrosine residues in its intracellular kinase domain, which further activates downstream transduction cascades, *e.g.* PI3K/AKT, MAPK/ERK and PLCγ1/PKC to exert cell proliferation and differentiation effect [2].

Signal transduction of EGFR is ordinarily under intimate control in human beings. However, tumor patients tend to display deregulated EGFR activity, mostly due to point mutations, exon 8 deletion or gene amplification [3–5]. Abnormal enhancement of EGFR activity represents a carcinogenesis initiator. In this context, the enormous relevance of anti-EGFR strategy *e.g*. small molecule tyrosine kinase inhibitors (TKI) gefitinib or monoclonal antibodies panitumumab and their clinical implication gained great success in the past years [6].

Besides functioning as carcinogenesis initiator, excessive EGFR activity is also considered to affect subsequent malignant development. Despite its unambiguous role as oncogene, the documentation of its clinical relevance is surprisingly heterogeneous in the scientific literature [7]. In the present investigation, we estimated the association of EGFR with clinical outcomes and pathological parameters at both mRNA and protein levels. We assessed *EGFR* mRNA expression and its correlation with overall survival (OS), TNM stage and grade of patients from 30 datasets covering 15 cancer types and compared 30 studies in this regard. We also performed immunohistochemical analysis on 502 human cases covering 27 tumor types and studied the correlation between EGFR protein expression and clinical outcomes or pathological characteristics corresponding to membranous and cytoplasmic or nuclear expression pattern as explanatory variable due to the fact that granular EGFR expression in the nucleus has been described as a factor of resistance to chemo- and radiotherapy [8–10]. Here, we integrated this information and considered, how it might be best applied for clinical routine diagnosis.

## RESULTS

### Correlation of *EGFR* mRNA expression and clinical outcomes

**Figure 1:**
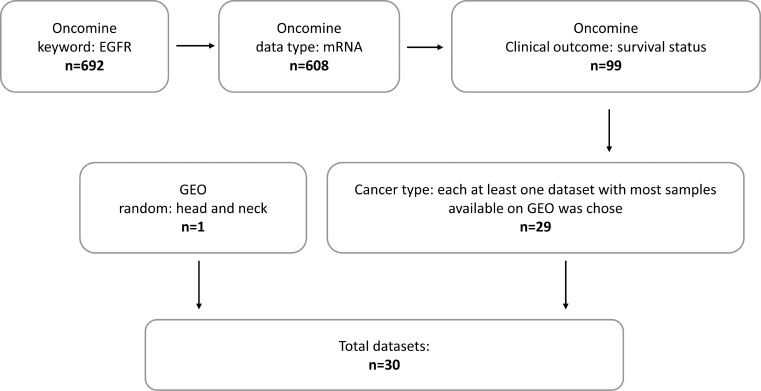
Filter flow for datasets screen

Thirty datasets were screened with filters in the Oncomine database. The filter flow is shown in Figure 1. Among 30 datasets (Tables 1–3), 23 datasets (=76.7%) did not show any significant association between *EGFR* mRNA level and clinical outcome or pathological characteristics of patients, except datasets GSE22226 and GSE10846, which showed significant associations between high *EGFR* mRNA expression levels and poor overall survival (cutoff mean, *p* = 0.03; cutoff mean, *p* = 0.03; respectively) (Table 1). However, adverse effects were documented in datasets GSE4412 and GSE15081 with statistical significance (cutoff median = mean, *p* = 0.02; cutoff median, probe AGhsB031519, *p* = 0.04), which indicated that high *EGFR* mRNA expression level was correlated with better overall survival.

**Table 1:** Correlation of EGFR mRNA expression and overall survival

Cancer type	GEO accession	Jetset probe	OS (*p* value)	
			Median	Mean
Bladder	GSE13507	ILMN_1696521	0.47	0.25
Brain	GSE7696	211607_x_at	0.12	0.28
	GSE4271	211607_x_at	0.19	0.19
	GSE4412	211607_x_at	0.02^*^	0.02^*^
Breast	GSE22226	A_23_P215790	0.07	0.03^*^
	GSE20685	211607_x_at	0.72	0.54
Colorectal	GSE17536	211607_x_at	0.01	0.01
Gastric	GSE15081	AGhsA201212	0.52	0.32
		AGhsB031519	0.04^*^	0.11
Head-Neck	GSE2379	1537_at	0.07	0.27
	GSE65858	ILMN_1696521	0.88	0.28
Leukemia	GSE12417	211607_x_at	0.17	0.14
Liver	GSE10186	DAP2_6059	0.12	0.08
	GSE364	NM_005228	0.33	0.28
Lung	GSE19188	211607_x_at	0.93	0.46
	GSE31210	211607_x_at	0.64	0.96
	GSE5123	X00588	0.50	0.61
	GSE4573	211607_x_at	0.22	0.27
Lymphoma	GSE4475	211607_x_at	0.53	0.76
	GSE10846	211607_x_at	0.08	0.03^*^
Melanoma	GSE8401	211607_x_at	0.64	0.47
	GSE2658	211607_x_at	0.59	0.52
	GSE19234	211607_x_at	0.97	0.60
Ovarian	GSE26712	211607_x_at	0.65	0.65
	GSE9899	211607_x_at	0.68	0.84
	GSE14764	211607_x_at	0.93	0.66
Pancreas	GSE17891	211607_x_at	0.95	0.89
Prostate	GSE6919	1537_at	0.67	0.67
	GSE10645	GI_29725608-S	0.43	0.51
Renal	GSE3538	AA234715	0.59	0.26
		W48712	0.20	0.20
		H80438	0.94	0.99

*P* value < 0.05 was labeled with asterisk mark. OS, overall survival. Median, group EGFR mRNA expression as “high” and “low” by median. Mean, group EGFR mRNA expression as “high” and “low” by mean.

Regarding tumor grade, datasets GSE5206 and GSE3538 showed a significant correlation between high *EGFR* mRNA expression and poor differentiation (cutoff median, *p* = 0.03; cutoff median = mean, *p* = 0.02; respectively) (Table 2). Conversely, dataset GSE4412 indicated a conflicting trend (cutoff median = mean, *p*=0.02). In addition, dataset GSE15081 conveyed a trend for association of *EGFR* mRNA with N stage, GSE3538 with grade (Tables 2 and 3).

**Table 2:** Correlation of EGFR mRNA expression and grade

Cancer type	GEO accession	Jetset probe	Grade (*p* value)	
			Median	Mean
Bladder	GSE13507	ILMN_1696521	0.86	0.81
Brain	GSE4271	211607_x_at	0.35	0.35
	GSE4412	211607_x_at	0.02^*^	0.02^*^
Breast	GSE22226	A_23_P215790	0.06	0.05
Colorectal	GSE17536	211607_x_at	0.06	0.10
	GSE5206	211607_x_at	0.03^*^	0.08
Gastric	GSE15081	AGhsA201212	0.08	0.17
		AGhsB031519	0.64	0.23
Head-Neck	GSE2379	1537_at	0.54	0.63
Liver	GSE364	NM_005228	0.19	0.16
Lung	GSE5123	X00588	0.38	0.08
	GSE4573	211607_x_at	0.46	0.42
Ovarian	GSE9899	211607_x_at	0.05	0.02^*^
	GSE14764	211607_x_at	0.83	0.85
Pancreas	GSE17891	211607_x_at	0.37	0.85
Renal	GSE3538	AA234715	0.36	0.28
		W48712	0.02^*^	0.02^*^
		H80438	0.34	0.36

*P* value < 0.05 was labeled with asterisk mark. Median, group EGFR mRNA expression as “high” and “low” by median. Mean, group EGFR mRNA expression as “high” and “low” by mean.

**Table 3:** Correlation of EGFR mRNA expression and TNM stage

Cancer type	GEO accession	Jetset probe	T (*p* value)		N (*p* value)		M (*p* value)	
			Median	Mean	Median	Mean	Median	Mean
Bladder	GSE13507	ILMN_1696521	0.56	0.47	0.08	0.31	0.66	0.53
Breast	GSE22226	A_23_P215790	0.29	0.26				
	GSE20685	211607_x_at	0.92	0.98	0.59	0.60	0.48	0.14
Colorectal	GSE5206	211607_x_at	0.63	0.78	0.68	0.59	0.73	0.62
Gastric	GSE15081	AGhsA201212			0.02^*^	0.10		
		AGhsB031519			0.35	0.97		
Head-Neck	GSE2379	1537_at	0.25	0.24	0.55	0.48		
	GSE65858	ILMN_1696521	0.26	0.11	0.13	0.09	0.25	0.09
Liver	GSE364	NM_005228					0.46	0.73
Lung	GSE5123	X00588			0.63	0.65	0.23	0.12
	GSE4573	211607_x_at	0.56	0.45	0.60	0.38		
Melanoma	GSE8401	211607_x_at	0.32	0.25	0.38	0.73	0.12	0.08
Pancreas	GSE17891	211607_x_at	0.97	0.46	0.87	0.42		
Prostate	GSE6919	1537_at	0.32	0.32	0.61	0.61		
	GSE10645	GI_29725608-S	0.34	0.28	0.84	0.51		

*P* value < 0.05 was labeled with asterisk mark. T, N and M represented T stage, N stage and M stage, respectively. Median, group EGFR mRNA expression as “high” and “low” by median. Mean, group EGFR mRNA expression as “high” and “low” by mean.

Since *EGFR* mRNA expression did not correlate with survival times of patients, we were interested to analyze, whether or not EGFR protein expression was of prognostic value.

### Survey of immunohistochemical studies

Thirty studies filtered with following keywords “EGFR”, “expression”, “predictor”, “biomarker” and “prognosis/prognostic” were included in our survey (Table 4). Eighteen studies (=60%) revealed that high EGFR protein expression significantly correlated with poor clinical outcome parameters, *e.g.* overall survival (OS), progression-free survival (PFS), disease-free survival (DFS), as well as poor pathological characteristics, *e.g.* TNM stage, grade or overall stage of patients. The other studies claimed no significant correlations.

**Table 4:** Survey of immunohistochemical studies

Citation	OS	PFS	DFS	T	N	M	Grade	Stage
J. Tol *et al*., 2010	0.210	0.260						
E. Despierre *et al*., 2015	0.273	0.835						
W. Hwangbo *et al*., 2013	NS							
D. Dionysopoulos *et al*., 2013	NS		NS					
D. Swinson *et al*., 2004	0.720							
F. Hirsch *et al*., 2003	0.220			0.680	0.070			0.170
J.-P. Spano *et al*., 2005	0.780			0.006^*^	0.120	0.880	0.590	
J. Lee *et al*., 2002	NS			0.270			0.390	
J. McKay *et al*., 2002	0.230				0.800		0.014	NS
F. Projetti *et al*., 2013	NS	NS						
L. Dova *et al*., 2007	NS							
A. Ema *et al*., 2015	0.039^*^			0.400	0.036^*^			0.012^*^
A. gatsuma *et al*., 2015	<0.001^*^			<0.001^*^	<0.001^*^			<0.001^*^
A. Hyogotani *et al*., 2012	0.019^*^						0.004^*^	0.001^*^
S. Wheeler *et al*., 2012		0.019^*^						
M. Parvin *et al*., 2016				0.480	0.067	0.856	0.270	
G. Lazaridis *et al*., 2014	0.016^*^							
H. Park *et al*., 2014			0.743	0.388	0.300		0.331	0.018^*^
I. Kallel *et al*., 2012	0.004^*^			0.041^*^			0.038^*^	
C.-W. Huang *et al*., 2013	<0.001^*^		<0.001^*^	0.531	0.755		0.028^*^	0.928
W. Jia *et al*., 2016	0.035^*^		0.046^*^	0.022^*^	0.000^*^		0.322	0.000^*^
C. Hedner *et al*., 2016	0.016^*^			0.712	0.917	0.299	0.924	
A. Atmaca *et al*., 2012	0.463	0.185						
A. Gröbe *et al*., 2014		0.830		0.202	0.024^*^		0.130	
P. Zhang *et al*., 2015	0.046^*^			0.005^*^	0.278			0.001^*^
N. Bassullu *et al*., 2012	NS						0.039^*^	
M. Katurić *et al*., 2014	0.022^*^							
A. Noske *et al*., 2011	0.002^*^							
D. Weber *et al*., 2012		0.050^*^						
G. Dorđević *et al*., 2012	0.046^*^			0.354				

Abbreviations: OS, overall survival; PFS, progression free survival; DFS, disease free survival; NS, not significant, but the article did not provide exact data; *P* value < 0.05 was labeled with asterisk mark.

Compared to rather poor prognostic value *EGFR* mRNA expression, EGFR protein expression was of superior utility. Likewise, more significant associations with pathological characteristics were observed.

### Correlation of EGFR protein expression and pathological parameters

To validate whether EGFR protein expression and specifically its expression pattern as mcEGFR or nEGFR may provide paired associations with pathological characteristics, we conducted immunohistochemistry on a total number of 502 cases covering 27 tumor types.

**Figure 2:**
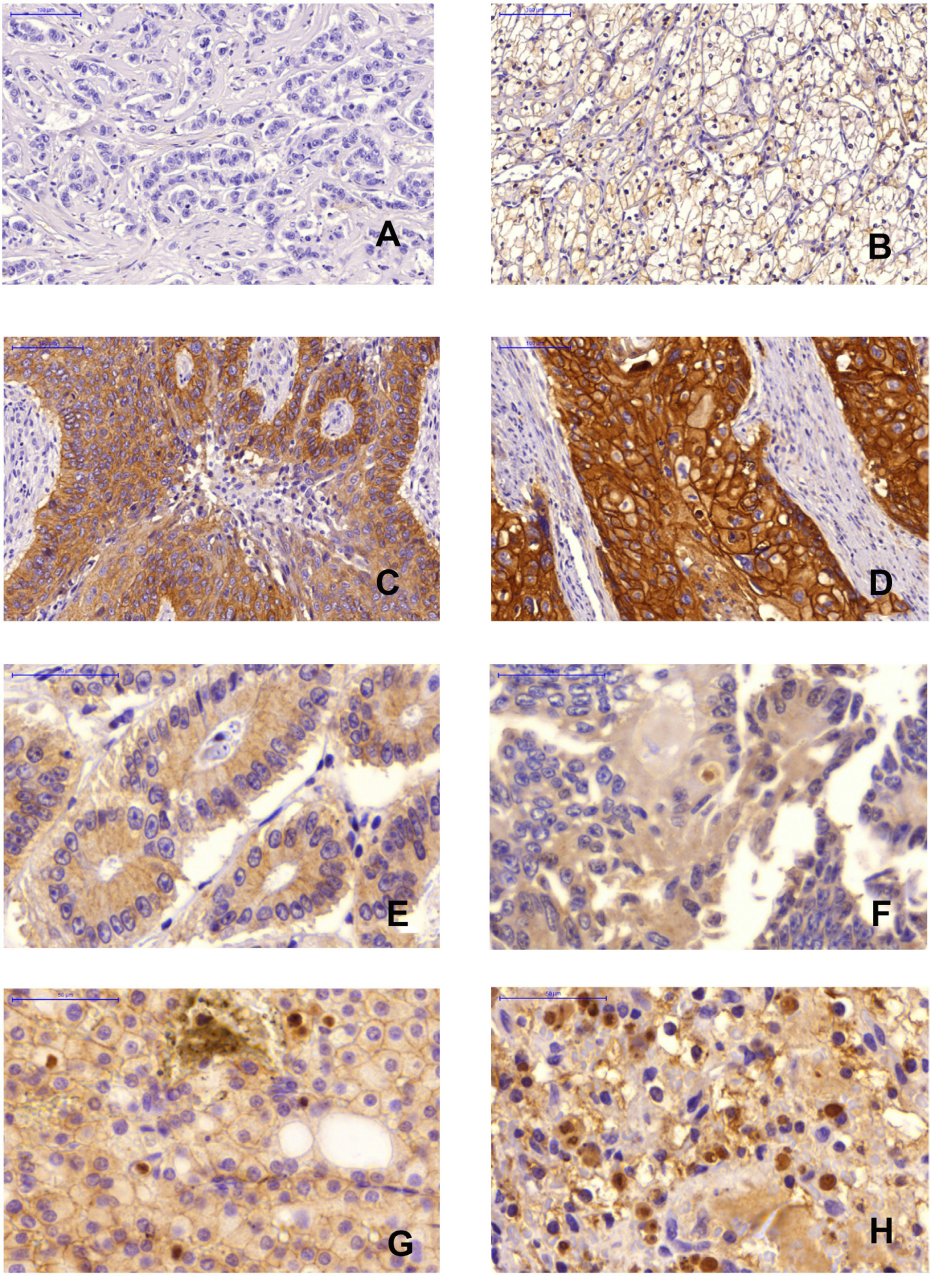
Immunohistochemical staining (**A**), Negative mcEGFR, breast tumor, 20×magnification; (**B**), Weak mcEGFR, kidney tumor, 20×magnification; (**C**), Moderate mcEGFR, lung tumor, 20×magnification; (**D**), Strong mcEGFR, esophagus tumor, 20×magnification; (**E**), Negative nEGFR, colon tumor, 40×magnification; (**F**), Weak nEGFR, colon tumor, 40×magnification; (**G**), Moderate nEGFR, kidney tumor, 40×magnification; (**H**), Strong nEGFR, kidney tumor, 40×magnification.

Among all cases, the frequency of negative, weak, moderate and strong staining was 36.25%, 30.08%, 27.89% and 5.78% for mcEGFR, while 48.24%, 26.13%, 15.08%, 10.55% of the tumors revealed nEGFR (Figure 2).

**Figure 3:**
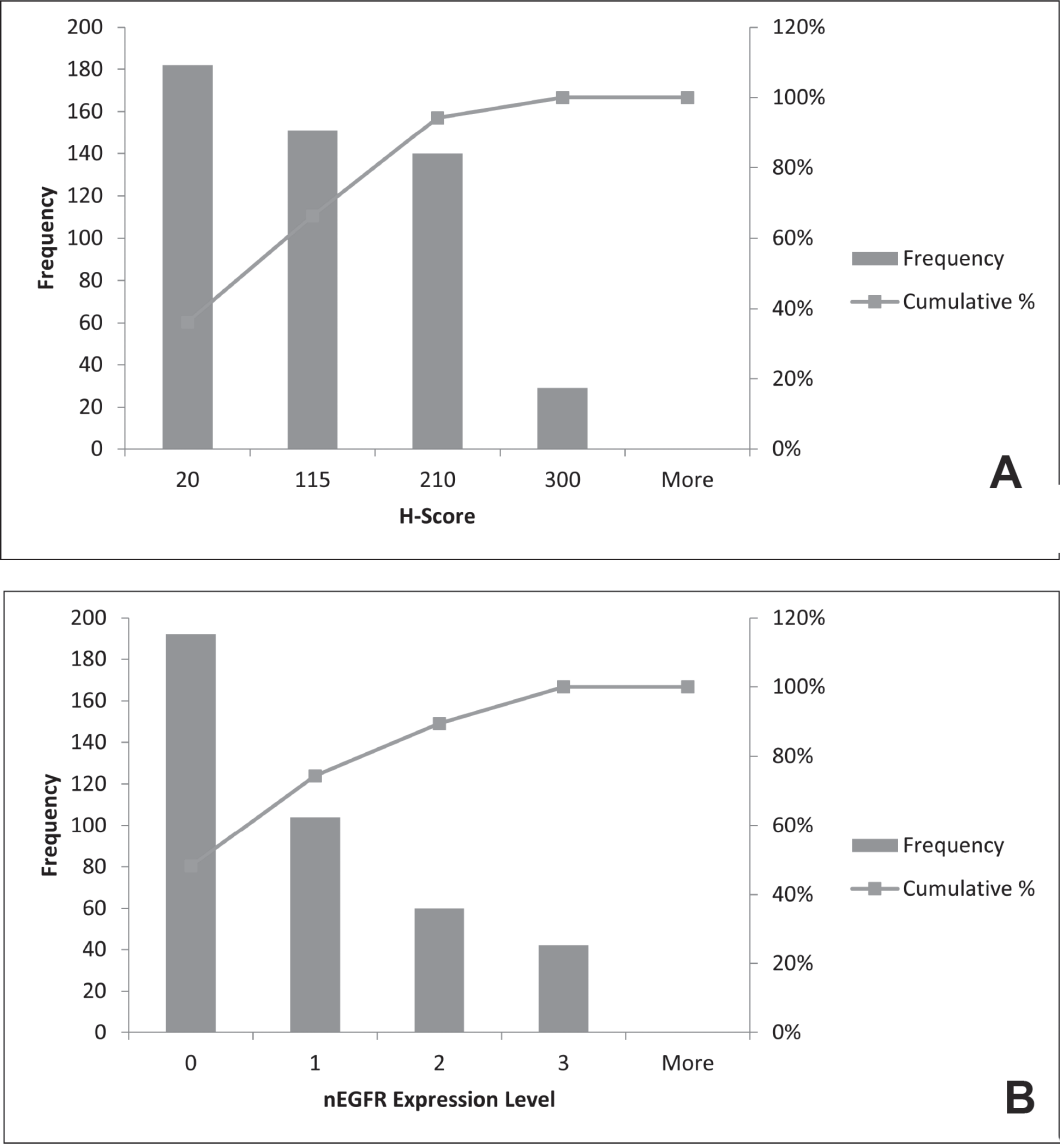
Distribution of mcEGFR and nEGFR among all tumor types (**A**), histogram of H-score, as indicator of mcEGFR expression, distribution among all 502 biopsies. (**B**), histogram of nEGFR distribution among all 398 biopsies. 0, 1, 2 and 3 on x-axis in histogram of nEGFR level indicated negative, weak, moderate and strong expression respectively.

Based on our investigation, higher expression of both mcEGFR and nEGFR was accompanied with lower occurrence (Figure 3). In other words, extreme high EGFR expression regardless of membrane-bound or nuclear expression patterns was rather rare among the tumors investigated.

**Figure 4:**
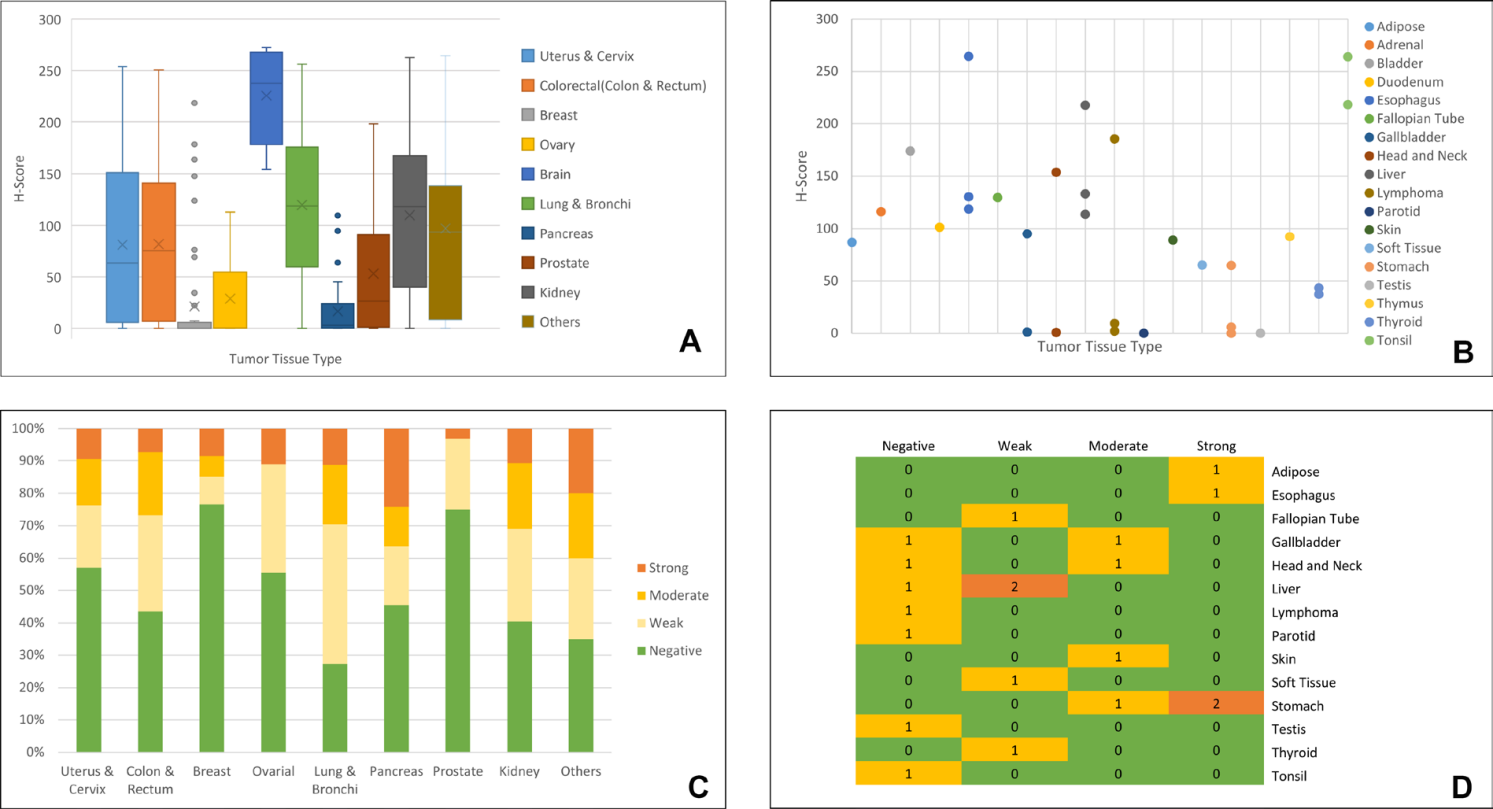
Distribution of EGFR in different tumor tissue types (**A**), H-score, as indicator of mcEGFR expression, distribution in different tumor types. All the tumor types comprising less than 5 cases were grouped as “others”. Tissue types were color coded as shown in legend. (**B**), H-score distribution among “others”. In this figure, cases of each tumor type were less than 5. Plot was drawn according to H-score and tumor types. Tissue types were color coded as shown in legend. (**C**), Distribution of nEGFR among different tumor types. nEGFR levels were classified as “Negative”, “Weak”, “Moderate” and “Strong” and each level was coded with green, light yellow, yellow and orange respectively. (**D**), nEGFR expression among tumor types with less than 5 cases. Heat map was drawn according to nEGFR level and tissue type. 3-Color scale indicated frequency of nEGFR expression where green showed 0 case, yellow showed 1 case while orange showed 2 cases. Detailed information about “others” refers to Supplementary Table 2.

Furthermore, we identified the distribution of mcEGFR and nEGFR expression in different tumor types (Figure 4). As shown in Figure 4A, mcEGFR was highly expressed in brain tumors followed by lung tumors. Compared to lung tumors, the expression in brain tumors tend to be more intensive if the whisker range was put into consideration. Uterus, colorectal and kidney tumors expressed mcEGFR in a similar manner. Breast, ovary, pancreas and prostate tumors revealed comparatively low expression levels. Noticeably, there were a few cases of breast tumors with strong mcEGFR expression, which exceeded the whisker range. Tumor types comprising less than 5 cases were classified as “others” (Figure 4B), among which fallopian tube tumor ranked top while parotid and testis ranked the lowest. However, the results could not provide accurate information due to limited case number. In the case of nEGFR, brain tumors were excluded from analysis due to the difficulty in determining nEGFR in this tumor entity. By contrast, nEGFR was frequently found in lung tumors followed by kidney, colorectum, pancreas, ovary and uterus, respectively (Figure 4C). In addition, stomach tumors also expressed high nEGFR (Figure 4D). However, nEGFR expression in breast and prostate was comparatively rare.

To explore the relationship between mcEGFR and nEGFR, we performed independent *t*-tests with negative or positive expression of nEGFR as grouping variable. Furthermore, we categorized the H-score as described above into four levels. Pearson's χ2-test was applied to assess the independence between H-score levels and nEGFR levels (Table 5). The result provided a compelling argument that mcEGFR and nEGFR are dependent factors (*p* < 0.001). Besides, there was a significant difference of H-score mean value between negative nEGFR and positive nEGFR groups (*p* < 0.001) which indicated cases harboring negative nEGFR also showed lower mcEGFR expression compared to positive nEGFR cases.

**Table 5:** Correlation between mcEGFR and nEGFR

mcEGFR No. patients (% within H-score)									
		Independent *t*-test		Pearson's χ^2^-test					
		Mean	*P* Value		Negative	Weak	Moderate	Strong	*P* Value
nEGFR	Negative	37.416		Negative	115 (70.12)	54 (46.55)	21 (21.43)	2 (10.00)	
	Positive	101.528		Weak	23 (14.02)	35 (30.17)	40 (40.82)	6 (30.00)	
				Moderate	12 (7.32)	18 (15.52)	22 (22.45)	8 (40.00)	
			8.44E–11^*^	Strong	14 (8.54)	9 (7.76)	15 (15.31)	4 (20.00)	2.85E–13^*^

*P* value < 0.05 was labeled with asterisk mark.

**Figure 5:**
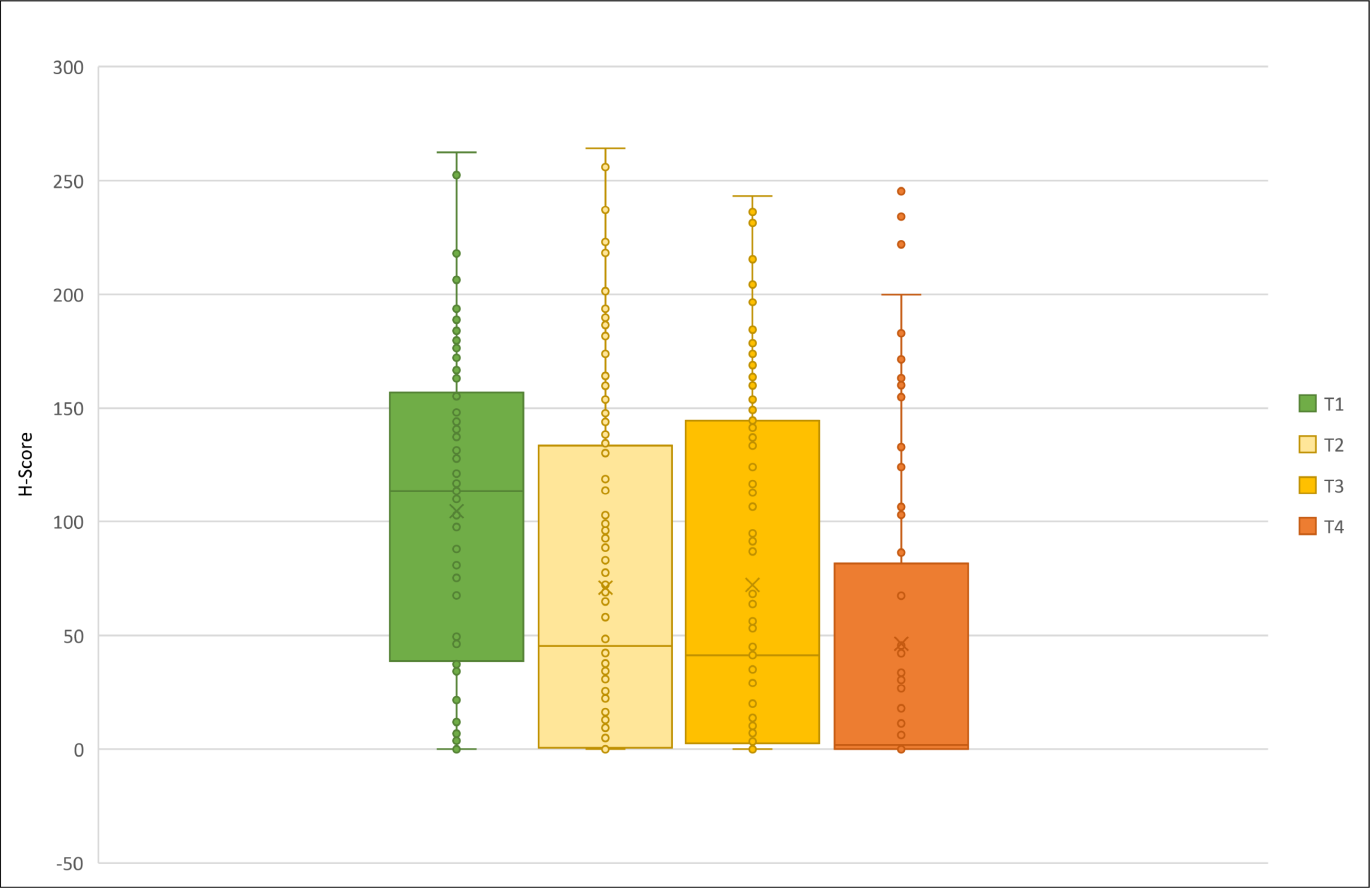
Correlation between H-score and T stage T stage was color coded as green represented T1 stage, light yellow T2, yellow T3 while orange T4.

To further explore the correlation of EGFR protein expression and pathological characteristics, we firstly run ANOVA mean comparison test for mcEGFR H-score, TNM stage and grade, respectively. Then, we used Pearson's χ2-test to determine the independence of H-score as negative and positive groups with TNM stage and grade, respectively. Unexpectedly, there was an adverse association between mcEGFR and T stage as mean comparison (Figure 5, *p* < 0.001). In addition, H-score and T stage were dependent in an adverse manner as well (*p* < 0.001). Moreover, positive mcEGFR was associated with low grade (*p* = 0.027) in Pearson's χ2-test. The same trend was also found in one-way ANOVA mean comparison test but without significance (*p* = 0.233). However, no significant difference was found among any other pathological parameters. Neither were any dependent relationships in between these parameters (Table 6). Interestingly, nEGFR revealed consistent results that its expression and T stage was adversely dependent (*p* = 0.004) by Pearson's χ2-test (Table 6).

**Table 6:** Correlation of EGFR protein expression and pathological characteristics

		mcEGFR No. patients (% within pathological parameters)					nEGFR No. patients (% within pathological parameters)		
			One way ANOVA /independent *t*-test		Pearson's χ^2^-test			Pearson's χ^2^-test		
		Mean	*P* value	Negative	Positive	*P* Value	Negative	Positive	*P* value
T Stage	T1	104.460		14(15.7)	75(84.3)		26(39.4)	40(60.6)	
	T2	70.918		55(41.7)	77(58.3)		58(54.2)	49(45.8)	
	T3	72.144		46(40.0)	69(60.0)		40(40.4)	59(59.6)	
	T4	46.390	3.18E-05^*^	37(61.7)	23(38.3)	2.19E-07^*^	32(68.1)	15(31.9)	0.004^*^
N Stage	N0	63.121		100(47.2)	112(52.8)		93(55.4)	75(44.6)	
	N1	67.407		36(46.8)	41(53.2)		31(44.3)	39(55.7)	
	N2	73.402	0.812	5(29.4)	12(70.6)	0.365	7(63.6)	4(36.4)	0.224
M Stage	M0	72.587		133(39.9)	200(60.1)		134(50.2)	133(49.8)	
	M1	62.534	0.606	11(44.0)	14(56.0)	0.690	10(50.0)	10(50.0)	0.987
Grade	Low	81.664		76(32.3)	159(67.7)		95(50.8)	92(49.2)	
	High	70.133	0.233	64(43.5)	83(56.5)	0.027^*^	50(42.4)	68(57.6)	0.151

*P* value < 0.05 was labeled with asterisk mark. G0 and N3 cases were excluded for analysis. Well differentiated to moderate differentiated cases were grouped as low grade while moderate-to-poorly differentiated to poorly differentiated cases were grouped as high grade.

## DISCUSSION

EGFR is well-known as oncogenic signal regulating proliferation apoptosis and differentiation and thereby contributes to carcinogenesis. The development of specific small molecules and antibodies targeted to EGFR represents an attractive clinical implementation [11, 12].

The reasons for EGFR overexpression are related with *EGFR* gene amplification, receptor-activating mutations, or deficiency of negative regulatory mechanisms [13]. Here, we investigated prognostic value of *EGFR* mRNA expression by mining the data deposited in the GEO and Oncomine databases. Although there are studies revealing that high *EGFR* mRNA [13–18] or even gene copy number [19] was correlated with poor clinical outcomes or pathological characteristics, a more systematic evaluation of published studies did not validate the proposed impact of *EGFR* mRNA expression. The inconsistency partially may be attributed to the choice of the *EGFR* probe. Microarray chips normally provided several probes targeting the same gene. Expression intensity according to different probes can extraordinarily differ, which may even lead to completely opposite conclusions. We used the optimal probe for our analysis based on the concept of jetset probe [20], which means only those probes providing comparatively better overall specificity, coverage and robustness were chosen. Since no correlation was found based on mRNA expression, we assessed 30 independent studies assuming that EGFR protein expression might be a more promising prognostic factor than *EGFR* mRNA expression.

As demonstrated by elegant analyses, there exist two distinct patterns of EGFR expression. Upon stimulation with ligands, mcEGFR undergoes COPI-mediated retrograde trafficking from the Golgi apparatus to the endoplasmic reticulum. With the help of importin β1 and Sec61β, mcEGFR can be shuttled from outer nuclear membrane to inner nuclear membrane and finally released into nucleoplasm and become nEGFR [21, 22]. Therefore, we took one step further and investigated, whether protein expression patterns as membranous and cytoplasmic or nuclear expression would make a difference in regard of affecting clinical outcomes or pathological characteristics. Although it has been reported that once entered into nucleus, nEGFR functions in a manner distinct from its cytoplasmic membrane counterpart [9, 23, 24, 10, 25–27], we primarily focused on clarifying the relationship between mcEGFR and nEGFR. In the current study, we observed a clear positive correlation between mcEGFR and nEGFR (*p* < 0.001). Furthermore, both mcEGFR and nEGFR expressions were unexpectedly associated with T stage in an adverse manner (*p* < 0.001 and *p* = 0.004; respectively). Positive mcEGFR was related to well differentiation (*p* = 0.027). We also revealed the diverse distribution patterns of both mcEGFR and nEGFR within different tumor types.

Taken together, our results indicated that protein rather than mRNA expression reflects the prognostic value of EGFR. This may have important implications, since results based on *EGFR* expression obtained by mRNA microarray and next generation sequencing technologies may be less informative than those resulting from protein arrays or immunohistochemical analyses. Recently, the nuclear expression of EGFR came more into the focus of attention, which can be only monitored by methods based on protein visualization and localization. Furthermore, the fact that both mcEGFR and nEGFR expression was rather associated with low T stage and positive mcEGFR was related to low grade, thus high tissue differentiation, may imply that the oncogenic function of EGFR may be more related to nascent stages of carcinogenesis than to advanced and progressive tumors, which may as well explain at least partially the occurrence of secondary resistance against EGFR-directed therapy.

## MATERIALS AND METHODS

### Tumor cases

A total number of 502 formalin-fixed and paraffin-embedded tumor cases covering 27 tumor types have been obtained from different sources: Ovarian and endometrial carcinoma biopsies were provided by Prof. Jose Schneider and belong to the tumor banks of Hospital Universitario de Cruces, Bilbao, Spain and Hospital Universitario Valdecilla, Santander, Spain, respectively, and were to a large extent used in previous studies on oncogenic activation in gynecologic tumors [28, 29]. Relevant data and ethical approval by Wandsworth Ethics Committee (Wandsworth, UK, Ref: 08/H0803/3) regarding colon cancer has been published by us [30]. Further tumor biopsies have been obtained from Dr. Zahir Yassin (Tayba Cancer Centre, Khartoum, Sudan) with ethical approval from the National Medicines ans Poisons Board, Sudan (dated: September 20, 2015; Ref.: TQM/Pir-F/4). In addition, two tissue microarrays (TMAs) BC000119 (Biomax Inc., Derwood, USA) and T8235713 (Biocat, Heidelberg, Germany) were commercially available. Three further TMAs were provided by the Tissue Bank of the Institute of Pathology, University Medical Center, Mainz, Germany) with ethical approval from The Ethics Committee of the State Authorization Association for Medical Issues (*Landesärtzekammer)* Rheinland Pfalz (dated: March 22, 2018; Ref. 2018-13179). All patients gave informed consent prior to participation. All tumor cases information refers to Supplementary Table 1.

### Statistical evaluation of the GEO and Oncomine databases

*EGFR* mRNA expression data and corresponding overall survival time, TNM stage and grade information were obtained from the GEO (https://www.ncbi.nlm.nih.gov/geo/) and Oncomine (https://www.oncomine.org/) databases. Normalized and log-2 transformed *EGFR* mRNA expression values of jetset probes were further determined as “low” or “high” using both median and mean as the cut-off value. Thirty datasets covering 15 cancer types were analyzed for time-to-event distributions estimated with Kaplan–Meier curves with log-rank test as assessing significance method. Associations of *EGFR* mRNA expression level with pathological characteristics were determined by Pearson's χ2-test. The above mentioned statistical analyses were performed using IBM SPSS Statistics version 23 (IBM, USA). Statistical differences with *p*-values less than 0.05 were considered as significant.

### Search strategy

Thirty independent studies [14, 19, 31–58] based on immunohistochemical EGFR determination from Pubmed engine (https://www.ncbi.nlm.nih.gov/pubmed) were identified by combining the search terms “EGFR”, “expression”, “predictor”, “biomarker” and “prognosis/prognostic” for estimating EGFR protein expression and its correlation with clinical outcomes in comparison to analyses derived from the GEO and Oncomine databases based on mRNA expression.

### Immunohistochemistry and statistical application

Immunohistochemistry was performed on 502 biopsies using EGFR rabbit monoclonal antibody (Clone EP38Y; Thermo Fisher Scientific, Dreieich, Germany) as primary antibody. The staining procedure has been previously published by us [59]. Quantification of immunostainings was performed by using Panoramic Desk (3D Histotech Panoramic digital slide scanner, Budapest, Hungary). Membranous and cytoplasmic EGFR (mcEGFR) was quantified by MembraneQuant software by using H-Score. A minimum of each three representative areas per tumor were scanned and the mean values together with standard deviations were calculated. One-hundred-four cases were excluded for nuclear EGFR (nEGFR) analysis due to the limitation in distinguishing extremely positive mcEGFR and existence of nEGFR. The other 398 cases were manually graded regarding nEGFR expression.

We used one-way ANOVA to exert mean comparison of mcEGFR H-score within different cancer types, TNM stage and grade, respectively. Independent *t*-test was used to determine variation in distribution of mcEGFR H-score in nEGFR negative and positive groups. mcEGFR and nEGFR were further categorized into four degrees or negative and positive groups according to expression intensity. As to mcEGFR H-scores, values below 20 were grouped as negative; H-scores ranging from 20 to 115 as weakly positive, from 115 to 210 as moderate positive and above 210 as strongly positive. The later three groups were all considered as positive. The signal-to-noise cutoff of mcEGFR H-score was determined by H-score obtained from negative controls (omission of primary antibody during staining procedure). nEGFR was similarly grouped as negative, weak, moderate and strong positive immunostaining or as negative and positive groups. As categorical data, both mcEGFR and nEGFR and their association with pathological TNM stage and grade was assessed by Pearson‘s χ2-test. Above statistical analyses were performed by using IBM SPSS Statistics version 23 (IBM, USA). Statistical differences with *p*-values less than 0.05 were considered as significant. Noticeably, as to grade-relevant analyses, cases graded as G0 were excluded, well differentiated to moderate differentiated cases were grouped as low grade, while moderate-to-poorly differentiated to poorly differentiated cases were grouped as high grade.

## SUPPLEMENTARY MATERIALS TABLES





## Abbreviations

AKT: Protein kinase B
DFS: disease-free survival
EGF: epidermal growth factor
EGFR: epidermal growth factor receptor
ERK: extracellular signal–regulated kinase
HER: human epidermal growth factor receptor
MAPK: mitogen-activated protein kinase
mcEGFR: membranous and cytoplasmic epidermal growth factor receptor
nEGFR: nuclear epidermal growth factor receptor
OS: overall survival
PFS: progression-free survival
PI3K: Phosphatidylinositol-4: 5-bisphosphate 3-kinase
PKC: protein kinase C
PLCγ1: Phosphoinositide-specific phospholipase C
TGF-α: transforming growth factor α
TKI: tyrosine kinase inhibitor
TMA: Tissue Microarray
TRK: tyrosine kinase receptors.

## References

[1] Lemmon MA, Schlessinger J, Ferguson KM (2014). The EGFR family. Not so prototypical receptor tyrosine kinases. Cold Spring Harb Perspect Biol.

[2] Roskoski R (2014). The ErbB/HER family of protein-tyrosine kinases and cancer. Pharmacol Res.

[3] Gan HK, Kaye AH, Luwor RB (2009). The EGFRvIII variant in glioblastoma multiforme. J Clin Neurosci.

[4] Jia XF, Li J, Zhao HB, Liu J, Liu JJ (2015). Correlation of EGFR gene amplification with invasion and metastasis of non-small cell lung cancer. Genet Mol Res.

[5] Mitsudomi T (2014). Molecular epidemiology of lung cancer and geographic variations with special reference to EGFR mutations. Transl Lung Cancer Res.

[6] Song QB, Wang Q, Hu WG (2015). Anti-epidermal growth factor receptor monoclonal antibodies in metastatic colorectal cancer. A meta-analysis. World J Gastroenterol.

[7] Arteaga CL (2002). Epidermal growth factor receptor dependence in human tumors. More than just expression?. Oncologist.

[8] Brand TM, Iida M, Luthar N, Starr MM, Huppert EJ, Wheeler DL (2013). Nuclear EGFR as a molecular target in cancer. Radiother Oncol.

[9] Dittmann K, Mayer C, Paasch A, Huber S, Fehrenbacher B, Schaller M, Rodemann HP (2015). Nuclear EGFR renders cells radio-resistant by binding mRNA species and triggering a metabolic switch to increase lactate production. Radiother Oncol.

[10] Li C, Iida M, Dunn EF, Ghia AJ, Wheeler DL (2009). Nuclear EGFR contributes to acquired resistance to cetuximab. Oncogene.

[11] Efferth T (2012). Signal transduction pathways of the epidermal growth factor receptor in colorectal cancer and their inhibition by small molecules. Curr Med Chem.

[12] Kadioglu O, Cao J, Saeed ME, Greten HJ, Efferth T (2015). Targeting epidermal growth factor receptors and downstream signaling pathways in cancer by phytochemicals. Target Oncol.

[13] Franovic A, Gunaratnam L, Smith K, Robert I, Patten D, Lee S (2007). Translational up-regulation of the EGFR by tumor hypoxia provides a nonmutational explanation for its overexpression in human cancer. Proc Natl Acad Sci U S A.

[14] Ema A, Waraya M, Yamashita K, Kokubo K, Kobayashi H, Hoshi K, Shinkai Y, Kawamata H, Nakamura K, Nishimiya H, Katada N, Watanabe M (2015). Identification of EGFR expression status association with metastatic lymph node density (ND) by expression microarray analysis of advanced gastric cancer. Cancer Med.

[15] Fujita H, Ohuchida K, Mizumoto K, Itaba S, Ito T, Nakata K, Yu J, Kayashima T, Hayashi A, Souzaki R, Tajiri T, Onimaru M, Manabe T (2011). High EGFR mRNA expression is a prognostic factor for reduced survival in pancreatic cancer after gemcitabine-based adjuvant chemotherapy. Int J Oncol.

[16] Hoffmann AC, Goekkurt E, Danenberg PV, Lehmann S, Ehninger G, Aust DE, Stoehlmacher-Williams J. EGFR (2013). FLT1 and heparanase as markers identifying patients at risk of short survival in cholangiocarcinoma. PLoS One.

[17] Matsubara J, Yamada Y, Nakajima TE, Kato K, Hamaguchi T, Shirao K, Shimada Y, Shimoda T (2008). Clinical significance of insulin-like growth factor type 1 receptor and epidermal growth factor receptor in patients with advanced gastric cancer. Oncology.

[18] Nymoen DA, Hetland Falkenthal TE, Holth A, Ow GS, Ivshina AV, Tropé CG, Kuznetsov VA, Staff AC, Davidson B (2015). Expression and clinical role of chemoresponse-associated genes in ovarian serous carcinoma. Gynecol Oncol.

[19] Park HS, Jang MH, Kim EJ, Kim HJ, Lee HJ, Kim YJ, Kim JH, Kang E, Kim SW, Kim IA, Park SY (2014). High EGFR gene copy number predicts poor outcome in triple-negative breast cancer. Mod Pathol.

[20] Li Q, Birkbak NJ, Gyorffy B, Szallasi Z, Eklund AC (2011). Jetset. Selecting the optimal microarray probe set to represent a gene. BMC Bioinformatics.

[21] Faria JA, de Andrade C, Goes AM, Rodrigues MA, Gomes DA (2016). Effects of different ligands on epidermal growth factor receptor (EGFR) nuclear translocation. Biochem Biophys Res Commun.

[22] Lo HW, Ali-Seyed M, Wu Y, Bartholomeusz G, Hsu SC, Hung MC (2006). Nuclear-cytoplasmic transport of EGFR involves receptor endocytosis, importin beta1 and CRM1. J Cell Biochem.

[23] Han W, Carpenter RL, Cao X, Lo HW (2013). STAT1 gene expression is enhanced by nuclear EGFR and HER2 via cooperation with STAT3. Mol Carcinog.

[24] Huang WC, Chen YJ, Li LY, Wei YL, Hsu SC, Tsai SL, Chiu PC, Huang WP, Wang YN, Chen CH, Chang WC, Chang WC, Chen AJ (2011). Nuclear translocation of epidermal growth factor receptor by Akt-dependent phosphorylation enhances breast cancer-resistant protein expression in gefitinib-resistant cells. J Biol Chem.

[25] Liu K, Jiang T, Ouyang Y, Shi Y, Zang Y, Li N, Lu S, Chen D (2015). Nuclear EGFR impairs ASPP2-p53 complex-induced apoptosis by inducing SOS1 expression in hepatocellular carcinoma. Oncotarget.

[26] Lo HW, Hsu SC, Ali-Seyed M, Gunduz M, Xia W, Wei Y, Bartholomeusz G, Shih JY, Hung MC (2005). Nuclear interaction of EGFR and STAT3 in the activation of the iNOS/NO pathway. Cancer Cell.

[27] Wang SC, Nakajima Y, Yu YL, Xia W, Chen CT, Yang CC, McIntush EW, Li LY, Hawke DH, Kobayashi R, Hung MC (2006). Tyrosine phosphorylation controls PCNA function through protein stability. Nat Cell Biol.

[28] González-Rodilla I, Aller L, Llorca J, Muñoz AB, Verna V, Estévez J, Schneider J (2013). The E-Cadherin expression vs. tumor cell proliferation paradox in endometrial cancer. Anticancer Res.

[29] Schneider J, Centeno M, Jimenez E, Rodriguez-Escudero FJ, Romero H (1997). Correlation of MDR1 expression and oncogenic activation in human epithelial ovarian carcinoma. Anticancer Res.

[30] Krishna S, Ganapathi S, Ster IC, Saeed ME, Cowan M, Finlayson C, Kovacsevics H, Jansen H, Kremsner PG, Efferth T, Kumar D (2015). A Randomised, Double Blind, Placebo-Controlled Pilot Study of Oral Artesunate Therapy for Colorectal Cancer. EBioMedicine.

[31] Atmaca A, Werner D, Pauligk C, Steinmetz K, Wirtz R, Altmannsberger HM, Jäger E, Al-Batran SE (2012). The prognostic impact of epidermal growth factor receptor in patients with metastatic gastric cancer. BMC Cancer.

[32] Bassullu N, Turkmen I, Dayangac M, Yagiz Korkmaz P, Yasar R, Akyildiz M, Yaprak O, Tokat Y, Yuzer Y, Bulbul Dogusoy G (2012). The Predictive and Prognostic Significance of c-erb-B2, EGFR, PTEN, mTOR, PI3K, p27, and ERCC1 Expression in Hepatocellular Carcinoma. Hepat Mon.

[33] Despierre E, Vergote I, Anderson R, Coens C, Katsaros D, Hirsch FR, Boeckx B, Varella-Garcia M, Ferrero A, Ray-Coquard I, Berns EM, Casado A, Lambrechts D, Jimeno A, European Organisation for Research and Treatment of Cancer-Gynaecological Cancer Group (EORTC-GCG), and Groupe d'Invest igateurs Nationaux pour les Etudes des Cancers de l’Ovaire (GINECO), and Austrian Arbeitsgemeinschaft für Gynäkologische Onkologie (A-AGO), and National Cancer Research Institute (NCRI), and Australia New Zealand Gynaecological Oncology Group (ANZGOG), and Mario Negri Gynecologic Oncology group (MaNGO) (2015). Epidermal Growth Factor Receptor (EGFR) Pathway Biomarkers in the Randomized Phase III Trial of Erlotinib Versus Observation in Ovarian Cancer Patients with No Evidence of Disease Progression after First-Line Platinum-Based Chemotherapy. Target Oncol.

[34] Dionysopoulos D, Pavlakis K, Kotoula V, Fountzilas E, Markou K, Karasmanis I, Angouridakis N, Nikolaou A, Kalogeras KT, Fountzilas G (2013). Cyclin D1, EGFR, and Akt/mTOR pathway. Potential prognostic markers in localized laryngeal squamous cell carcinoma. Strahlenther Onkol.

[35] Dorđević G, Matušan Ilijaš K, Hadžisejdić I, Maričić A, Grahovac B, Jonjić N (2012). EGFR protein overexpression correlates with chromosome 7 polysomy and poor prognostic parameters in clear cell renal cell carcinoma. J Biomed Sci.

[36] Dova L, Pentheroudakis G, Georgiou I, Malamou-Mitsi V, Vartholomatos G, Fountzilas G, Kolaitis N, Kitsiou E, Pavlidis N (2007). Global profiling of EGFR gene mutation, amplification, regulation and tissue protein expression in unknown primary carcinomas. To target or not to target?. Clin Exp Metastasis.

[37] Gröbe A, Eichhorn W, Fraederich M, Kluwe L, Vashist Y, Wikner J, Smeets R, Simon R, Sauter G, Heiland M, Blessmann M (2014). Immunohistochemical and FISH analysis of EGFR and its prognostic value in patients with oral squamous cell carcinoma. J Oral Pathol Med.

[38] Hedner C, Borg D, Nodin B, Karnevi E, Jirström K, Eberhard J (2016). Expression and Prognostic Significance of Human Epidermal Growth Factor Receptors 1 and 3 in Gastric and Esophageal Adenocarcinoma. PLoS One.

[39] Hirsch FR, Varella-Garcia M, Bunn PA, Di Maria MV, Veve R, Bremmes RM, Barón AE, Zeng C, Franklin WA (2003). Epidermal growth factor receptor in non-small-cell lung carcinomas. Correlation between gene copy number and protein expression and impact on prognosis. J Clin Oncol.

[40] Huang CW, Tsai HL, Chen YT, Huang CM, Ma CJ, Lu CY, Kuo CH, Wu DC, Chai CY, Wang JY (2013). The prognostic values of EGFR expression and KRAS mutation in patients with synchronous or metachronous metastatic colorectal cancer. BMC Cancer.

[41] Hwangbo W, Lee JH, Ahn S, Kim S, Park KH, Kim CH, Kim I (2013). EGFR Gene Amplification and Protein Expression in Invasive Ductal Carcinoma of the Breast. Korean J Pathol.

[42] Hyogotani A, Ito K, Yoshida K, Izumi H, Kohno K, Amano J (2012). Association of nuclear YB-1 localization with lung resistance-related protein and epidermal growth factor receptor expression in lung cancer. Clin Lung Cancer.

[43] Jia W, Wang W, Ji CS, Niu JY, Lv YJ, Zhou HC, Hu B (2016). Coexpression of periostin and EGFR in patients with esophageal squamous cell carcinoma and their prognostic significance. Onco Targets Ther.

[44] Kallel I, Khabir A, Boujelbene N, Abdennadher R, Daoud J, Frikha M, Aifa S, Sallemi-Boudawara T, Rebaï A (2012). EGFR overexpression relates to triple negative profile and poor prognosis in breast cancer patients in Tunisia. J Recept Signal Transduct Res.

[45] Katunarić M, Jurišić D, Petković M, Grahovac M, Grahovac B, Zamolo G (2014). EGFR and cyclin D1 in nodular melanoma. Correlation with pathohistological parameters and overall survival. Melanoma Res.

[46] Lazaridis G, Lambaki S, Karayannopoulou G, Eleftheraki AG, Papaspirou I, Bobos M, Efstratiou I, Pentheroudakis G, Zamboglou N, Fountzilas G (2014). Prognostic and predictive value of p-Akt, EGFR, and p-mTOR in early breast cancer. Strahlenther Onkol.

[47] Lee JC, Wang ST, Chow NH, Yang HB (2002). Investigation of the prognostic value of coexpressed erbB family members for the survival of colorectal cancer patients after curative surgery. Eur J Cancer.

[48] McKay JA, Murray LJ, Curran S, Ross VG, Clark C, Murray GI, Cassidy J, McLeod HL (2002). Evaluation of the epidermal growth factor receptor (EGFR) in colorectal tumours and lymph node metastases. Eur J Cancer.

[49] Nagatsuma AK, Aizawa M, Kuwata T, Doi T, Ohtsu A, Fujii H, Ochiai A (2015). Expression profiles of HER2, EGFR, MET and FGFR2 in a large cohort of patients with gastric adenocarcinoma. Gastric Cancer.

[50] Noske A, Schwabe M, Weichert W, Darb-Esfahani S, Buckendahl AC, Sehouli J, Braicu EI, Budczies J, Dietel M, Denkert C (2011). An intracellular targeted antibody detects EGFR as an independent prognostic factor in ovarian carcinomas. BMC Cancer.

[51] Parvin M, Sabet-Rasekh P, Hajian P, Mohammadi Torbati P, Sabet-Rasekh P, Mirzaei H (2016). Evaluating the Prevalence of the Epidermal Growth Factor Receptor in Transitional Cell Carcinoma of Bladder and its Relationship With Other Prognostic Factors. Iran J Cancer Prev.

[52] Projetti F, Durand K, Chaunavel A, Léobon S, Lacorre S, Caire F, Bessède JP, Moreau JJ, Coulibaly B, Labrousse F (2013). Epidermal growth factor receptor expression and KRAS and BRAF mutations. Study of 39 sinonasal intestinal-type adenocarcinomas. Hum Pathol.

[53] Spano JP, Lagorce C, Atlan D, Milano G, Domont J, Benamouzig R, Attar A, Benichou J, Martin A, Morere JF, Raphael M, Penault-Llorca F, Breau JL (2005). Impact of EGFR expression on colorectal cancer patient prognosis and survival. Ann Oncol.

[54] Swinson DE, Cox G, O'Byrne KJ (2004). Coexpression of epidermal growth factor receptor with related factors is associated with a poor prognosis in non-small-cell lung cancer. Br J Cancer.

[55] Tol J, Dijkstra JR, Klomp M, Teerenstra S, Dommerholt M, Vink-Börger ME, van Cleef PH, van Krieken JH, Punt CJ, Nagtegaal ID (2010). Markers for EGFR pathway activation as predictor of outcome in metastatic colorectal cancer patients treated with or without cetuximab. Eur J Cancer.

[56] Weber DC, Tille JC, Combescure C, Egger JF, Laouiti M, Hammad K, Granger P, Rubbia-Brandt L, Miralbell R (2012). The prognostic value of expression of HIF1α, EGFR and VEGF-A, in localized prostate cancer for intermediate- and high-risk patients treated with radiation therapy with or without androgen deprivation therapy. Radiat Oncol.

[57] Wheeler S, Siwak DR, Chai R, LaValle C, Seethala RR, Wang L, Cieply K, Sherer C, Joy C, Mills GB, Argiris A, Siegfried JM, Grandis JR, Egloff AM (2012). Tumor epidermal growth factor receptor and EGFR PY1068 are independent prognostic indicators for head and neck squamous cell carcinoma. Clin Cancer Res.

[58] Zhang P, Wu SK, Wang Y, Fan ZX, Li CR, Feng M, Xu P, Wang WD, Lang JY (2015). p53, MDM2, eIF4E and EGFR expression in nasopharyngeal carcinoma and their correlation with clinicopathological characteristics and prognosis. A retrospective study. Oncol Lett.

[59] Michaelsen FW, Saeed ME, Schwarzkopf J, Efferth T (2015). Activity of Artemisia annua and artemisinin derivatives, in prostate carcinoma. Phytomedicine.

