# Supplementary material for: Relationship between EGFR expression and subcellular localization with cancer development and clinical outcome
Source: Oncotarget. 2019 Mar 8;10(20):1918–31. doi: 10.18632/oncotarget.26727 (PMC6443015; doi:10.18632/oncotarget.26727)
Supplement: Supplementary file 1 [file oncotarget-10-1918-s001.pdf]

Relationship between EGFR expression and subcellular localization with cancer development and clinical outcome

SUPPLEMENTARY MATERIALS

Supplementary Table 1: Tumor cases information. See Supplementary_Table_1

Supplementary Table 2: Detailed information of other tumor types

Others		
**Tumor type**	**Patients (*n* = 30) for mcEGFR analysis**	**Patients (*n* = 20) for nEGFR analysis**
Head and Neck	2	2
Bladder	1	0
Esophagus	3	1
Duodenum	1	0
Fallopian Tube	1	1
Gall Bladder	2	2
Liver	3	3
Lymphoma	3	1
Parotid	1	1
Skin	1	1
Soft Tissue	1	1
Tonsil	2	1
Stomach	3	3
Testis	1	1
Thymus	1	0
Thyroid	2	1
Adrenal	1	0
Adipose	1	1
Sum	30	20
