# Supplementary material for: Relationship between EGFR expression and subcellular localization with cancer development and clinical outcome
Source: Oncotarget. 2019 Mar 8;10(20):1918–31. doi: 10.18632/oncotarget.26727 (PMC6443015; doi:10.18632/oncotarget.26727)
Supplement: Supplementary file 2 [file oncotarget-10-1918-s002.pdf]

Supplementary Table 1: Tumor cases information

**Variables**	**Characteristics of patiens (*n*=502) for mcEGFR analysis**		**Characteristics of patients (*n*=398) for nEGFR analysis**	
	**No. of patients**	**% of total patients**	**No. of patients**	**% of total patients**
**Age Range**	24-95			
**Gender**				
Female	157		132	
Male	264		205	
NA	81		61	
Sum	502		398	
**Tissue type**				
Uterus & Cervix	25	4.98%	21	5.28%
Colorectal(Colon & Rectum)	138	27.49%	108	27.14%
Breast	51	10.16%	47	11.81%
Ovary	11	2.19%	9	2.26%
Brain	6	1.20%	0	0.00%
Lung & Bronchi	47	9.36%	44	11.06%
Pancreas	42	8.37%	33	8.29%
Prostate	44	8.76%	32	8.04%
Kidney	108	21.51%	84	21.11%
Other	30	5.98%	20	5.03%
Cases in Analysis	**502**	100.00%	**398**	100.00%
Sum	502	100.00%	398	100.00%
**Pathologic T Stage**				
T1	89	17.73%	66	16.58%
T2	132	26.29%	107	26.88%
T3	115	22.91%	99	24.87%
T4	60	11.95%	47	11.81%
NA	106	21.12%	79	19.85%
Cases in Analysis	**396**	78.88%	**319**	80.15%
Sum	502	100.00%	398	100.00%
**Pathologic N Stage**				
N0	212	42.23%	168	42.21%
N1	77	15.34%	70	17.59%
N2	17	3.39%	11	2.76%
N3	1	0.20%	1	0.25%
NA	195	38.84%	148	37.19%
Cases in Analysis	**306**	60.96%	**249**	62.56%
Sum	502	100.00%	398	100.00%
**Pathologic M Stage**				
M0	333	66.33%	267	67.09%
M1	25	4.98%	20	5.03%
NA	144	28.69%	111	27.89%
Cases in Analysis	**358**	71.31%	**287**	72.11%

Sum	502	100.00%	398	100.00%
**Pathologic Grade**				
G0	16	3.19%	15	3.77%
G1	69	13.75%	55	13.82%
G1-G2(G1.5)	9	1.79%	8	2.01%
G2	157	31.27%	124	31.16%
G2-G3(G2.5)	5	1.00%	3	0.75%
G3	141	28.09%	115	28.89%
G4	1	0.20%	0	0.00%
NA	104	20.72%	78	19.60%
Cases in Analysis	**382**	76.10%	**305**	76.63%
Sum	502	100.00%	398	100.00%

Case n=1 were excluded for later analysis, except for G4. G0 cases were excluded for later analysis. NA, not available.
